# Explainable sequence-to-sequence GRU neural network for pollution forecasting

**DOI:** 10.1038/s41598-023-35963-2

**Published:** 2023-06-19

**Authors:** Sara Mirzavand Borujeni, Leila Arras, Vignesh Srinivasan, Wojciech Samek

**Affiliations:** 1 Department of Artificial Intelligence, Fraunhofer Heinrich Hertz Institute, 10587 Berlin, Germany; 2 Department of Electrical Engineering and Computer Science, Technische Universität Berlin, 10587 Berlin, Germany; 3 BIFOLD–Berlin Institute for the Foundations of Learning and Data, 10587 Berlin, Germany

**Keywords:** Environmental impact, Computer science

## Abstract

The goal of pollution forecasting models is to allow the prediction and control of the air quality. Non-linear data-driven approaches based on deep neural networks have been increasingly used in such contexts showing significant improvements w.r.t. more conventional approaches like regression models and mechanistic approaches. While such deep learning models were deemed for a long time as black boxes, recent advances in eXplainable AI (XAI) allow to look through the model’s decision-making process, providing insights into decisive input features responsible for the model’s prediction. One XAI technique to explain the predictions of neural networks which was proven useful in various domains is Layer-wise Relevance Propagation (LRP). In this work, we extend the LRP technique to a sequence-to-sequence neural network model with GRU layers. The explanation heatmaps provided by LRP allow us to identify important meteorological and temporal features responsible for the accumulation of four major pollutants in the air ($\text {PM}_{10}$, $\text {NO}_{2}$, $\text {NO}$, $\text {O}_{3}$), and our findings can be backed up with prior knowledge in environmental and pollution research. This illustrates the appropriateness of XAI for understanding pollution forecastings and opens up new avenues for controlling and mitigating the pollutants’ load in the air.

## Introduction

Air pollution is one of the major concerns threatening human life on earth. Emission of detrimental pollutants into the air causes far-reaching damages to living species and the environment. It is approximated that poor air quality annually leads to 3.7 million deaths worldwide, besides the indirect damage caused by destroyed crops and water resources (e.g., through acid rain)^1,2^. Given how harmful air pollution can be, it is important to monitor changes in the level of pollutants in the air, as well as forecast their concentrations to mitigate the effects of this problem.

Thanks to the ever-increasing number of pollution detectors and meteorological sensors installed in different areas, we are able to track fluctuations in pollutant loads, and to collect enough data for pollution forecasting. However, these data contain various spatio-temporal dependencies, and therefore, to enable an accurate prediction, complex models are needed which can capture the involved relationships. One such model, which was shown to perform very well in practice, is based on a sequence-to-sequence neural network with Gated Recurrent Units (GRU) layers^3^. The model consists of an encoder and a decoder modules fed with sequences of data from the past seven days, and has the ability to accurately forecast pollutant concentrations for up to 48 h by combining local sensor measurements with global weather data.

Although this sequence-to-sequence pollution forecasting model exhibits good prediction results in a practical setup, so far no investigation was conducted to analyze *how* it reaches its decisions, which would be desirable for increasing trust in its functionality. Indeed, it has been noticed that some predictors perform well on their training and test datasets, but fail in real-world deployments. The work by Lapuschkin et al.^4^ refers to these kinds of model behaviours as “Clever Hans” predictors. Clever Hans was a famous horse in the early twentieth century known for its ability to solve mathematical problems. However, as it turned out, the horse in fact arrived at the right answer by observing the reaction of the enquirer. Hence, the cleverness of the horse was a false claim and its predictions were not reliable at all. There are other examples showing that models sometimes decide based on the wrong criteria^5,6^. Therefore, verification and inspection of AI decisions is crucial to foster reliability and trust. Additionally, explaining the behaviour of the model and determining the contributions of the input features to the final decision could also lead to a more efficient selection of features or optimization of the model.

Our main focus in this work is to explain the predictions of the pollution forecasting sequence-to-sequence model with GRU layers as was proposed in Petry et al.^3^, using the XAI method of LRP^7^, in order to demonstrate the usefulness of XAI for this task and domain. To this end, we extend the popular LRP technique to GRU layers and to a sequence-to-sequence recurrent architecture. This extension allows us to gain insights into which sets of input variables are the most relevant for predicting high concentration levels of various pollutants.

For instance, in the case of the pollutant *NO* (nitrogen monoxide), we find that the morning traffic hours play an important role in predicting high levels of the pollution, which is in agreement with results from previous work that show that *NO* displays a strong dependency on diurnal source emissions^8^, while for predicting the load of the other pollutants we considered in our work the hour of the day is less important, and other meteorological factors as well as historical measurements are instead determinant. Besides, for the $\text{O}_3$ (ozone) pollutant concentration, we find that the high predictions rely on warm temperature and low humidity, which is also consistent with well-established domain knowledge about the central role of solar radiation for ozone formation^9,10^, as well as well as prior work suggesting that dry deposition via trees might also be involved in regulating the $\text{O}_3$ concentration^11^.

Along the way, to ensure the correctness of our implementation, we verified the LRP relevance conservation property^12^, as well as validated the LRP input features’ relevances using a toy task (similarly to Arras et al.^13^). For reproducibility of our results we make our code publicly available (https://github.com/Sara-mibo/LRP_EncoderDecoder_GRU). More broadly, our explainable GRU-based and sequence-to-sequence recurrent model can be employed on any other task or domain that relies on the same neural network components. Finally, we show that it is possible to simplify our pollution forecasting model’s structure by inspecting the LRP results. This adjustment speeds up the training and inference by reducing the model’s complexity with a negligible change in the prediction performance.

## Related work

### Pollution forecasting

Conventional approaches for pollution forecasting are based on numerical simulations taking into account the physical and chemical processes involved in the emission, diffusion and transport of air pollutants. These models are built upon specialized sub-modules that deal with the different pollution causes (such as natural and anthropogenic sources, chemical transformations, aerosol processes and microphysics, pollutant transport through wind and diffusion, dry and wet deposition), and they require sophisticated parameterizations^14^.

Alternative data-driven approaches for pollution forecasting, and more generally for Earth system science, have also been explored recently based on regression analysis, autoregressive statistical models and machine learning^15–17^. Among these models, non-linear models such as artificial neural networks improved the prediction accuracy over linear models, and compared to mechanistic approaches, they present the advantage of not requiring a precise knowledge of the underlying chemical and physical processes. However they typically necessitate a larger amount of historical data to be available^18–23^. Additionally, hybrid systems combining different methods, e.g. autoregressive statistical models with artificial neural networks^24^, have also been proposed^15^. Besides, in order to explicitly incorporate the spatial dependency between locations and the diffusion of pollutants into the model, some recent works used an averaging of data across neighboring locations as input^25^, or feed the data into the neural network model via a grid structure using a convolutional or a graph neural network^26,27^ (which bears some similarity with the tasks of video and motion prediction^16^). In our work we focus on a model that primarily consideres temporal data as input (and we modelize the spatial location as a static input feature), as this setup was found to reach sufficient performance in a real-world application^3^, and our main objective in the present work is to demonstrate the pertinence of XAI in the pollution forecasting domain and using a standard and widely-used neural network architecture for temporal data.

Among artificial neural networks, recurrent networks are particularly well-suited to modelize temporal data. Compared to feed-forward neural networks where one has to select the time window and averaging of the input variables, recurrent networks can directly receive the raw time series as input and learn the useful temporal dependencies from the training data^28^. LSTMs^29,30^ and GRUs^31–33^ are two popular such recurrent network architectures that further alleviate the vanishing gradient problem of vanilla recurrent nets through the introduction of memory cells and gating mechanisms, that allow them to learn longer-range temporal dependencies. For example in the field of hydrology, the standard LSTM model as well as novel LSTM variants, were used to predict the basin water discharge from rainfall measurements and other temporal meteorological variables (as well as static catchment attributes), and were shown to outperform high-quality basin-calibrated hydrological models, in particular for flood prediction^34–36^. On the task of pollution forecasting that we consider in the present work, the LSTM and GRU networks were already compared to one another and achieved similar prediction performance^3^, though the GRU model has less parameters, therefore we use a GRU-based model in our work.

Despite their high prediction performance, thus far artificial neural networks were not largely adopted in pollution forecasting due to their lack of interpretability^28,37,38^. Indeed, as opposed to regression-based models where one can readily obtain the contributions of environmental variables to the prediction task, neural networks are typically considered as black-boxes. Notwithstanding, in recent years several post-hoc XAI methods have been been proposed to inspect the decision process of neural networks: given a trained neural network, these methods are able to assign to each input variable a relevance score representing the contribution of that variable to a given prediction^5,39^. These methods not only facilitate transparency and trust in the model’s decision, they potentially also lead to new scientific insights and discoveries^40^.

### Explanation methods

Up to now a limited number of studies already applied such XAI techniques to explain the predictions of neural networks for pollution forecasting. For example in Elangasinghe et al.^41^ the authors used sensitivity analysis to identify the most important input variables for the prediction of $\text {NO}_{2}$ using a feed-forward neural network, and successively removed the least significant features from the input to arrive at a simpler model with similar performance. In García et al.^42^ the authors used the SHAP method to interpret the decisions of an LSTM that also predicts $\text {NO}_{2}$ concentration, and checked the compatibility of the results with previous intuitions and scientific domain knowledge. Another recent work by Yang et al.^43^ analyzed the contribution of inputs upon recurrent networks trained on different sets of variables for the prediction of $\text {PM}_{2.5}$ using the SHAP method. In our work we will employ the XAI technique of LRP and verify the consistency of the results with available domain knowledge, as well as validate our results intrinsically by using the LRP relevances to simplify our model’s architecture.

More generally, among the existing post-hoc XAI methods for neural networks, one can essentially identify three main approaches. One is based on the gradient of the prediction function w.r.t. the input variables and includes Saliency^44–46^, Gradient times Input^47^ and Integrated Gradients^48^. While gradients are easy to compute, they are known to be noisy and subject to high variance, this is why smoothing techniques based on random sampling such as SmoothGrad have been proposed^49^. However the introduced post-processing step induces additional hyperparameters and randomness in the explanation process^50^. Another line of approach which is also model-agnostic is based on solving an ad-hoc optimization problem to learn a local linear model using perturbed or masked versions of the original input, which includes the LIME^6^ and the Kernel SHAP^51^ methods. These sampling-based techniques are typically non-deterministic, expensive to compute, and it is unclear how to extend them to recurrent networks that present temporal dependencies in the input sequence, since their original formulations do not consider the order of the inputs^52^. Finally, another way of explaining decisions is based on the layer-wise decomposition of the prediction function and involves a custom backward propagation through the network that verifies an overall conservation principle. LRP^7,12^ and Deep Taylor Decomposition^53^ are prime examples of this approach.

The LRP explanation method presents the advantage of being fast to compute, since it requires only a single backward pass through the network, however it is not model-agnostic and requires a specific implementation for each novel type of neural network layer. Theoretically, the LRP propagation procedure can be justified via the mathematical framework of Deep Taylor Decomposition^53,54^. In terms of practical application, LRP already demonstrated its superiority over other XAI methods on convolution neural networks in the computer vision domain^4,50,55^, as well as on a targeted simulation in Geoscience inspired by remote sensing tasks where spatial patterns need to be extracted^56^. Its usefulness has also been demonstrated in the Earth science field, where it was able to identify physically meaningful patterns of climate variability using feed-forward networks trained on geospatial fields of sea surface temperature^57^. With the present work, we further showcase its suitability in the environmental domain for the task of pollution forecasting, using a sequence-to-sequence GRU-based neural network model.

The LRP technique was previously extended to recurrent networks such as LSTMs^58^, and the resulting explanations were evaluated against other existing XAI methods on toy tasks and in natural language processing^13,54,59^, where LRP was shown to deliver superior results. Further, this LRP extension to recurrent networks was successfully applied in the health domain for therapy prediction^60^ and in computer security for discovering vulnerability in source code^61^. A recent work also applied the technique to a recurrent network for $\text {PM}_{2.5}$ forecasting, where it helped identifying a subset of input variables that lead to a similar prediction performance when the model was re-trained only on this subset^62^.

Therefore, we will employ the LRP explanation technique to explain our pollution forecasting model^3^. To this end we adapt the method from Arras et al.^58^ for a GRU layer and to a sequence-to-sequence network architecture.

## Pollution forecasting task

### Dataset

We consider data collected from a total of 22 measuring stations located at the city of Stuttgart and the federal state of North Rhine-Westphalia (NRW) in Germany, during the years 2010 to 2020. These stations are located at different places including urban, suburban and rural areas with diverse emission sources. Table 1 represents the input features used for training our model, along with their source and unit. These input features include the concentration of four different pollutants, namely $PM_{10}$, *NO*, $\text{NO}_2$ and $\text{O}_3$ (which are also the pollutants which are being forecasted), six meteorological features (temperature, precipitation, sunshine, humidity, wind direction and speed), five temporal features (year, month, day, hour, working vs. work-free day), as well as one static feature representing the station number. The temporal resolution (i.e. the time interval between two measurements/features) used for the inputs and the prediction is 1 h. The resulting collected dataset has more than 1 million data sequences of length 216 h (corresponding to 9 days) , which are distributed randomly into train, validation, and test sets with the proportions of 10/12, 1/12, 1/12.

During the forecast, the measured meteorological input features are replaced by weather forecasts, and the measured pollutant concentrations by the corresponding predicted ones in previous time steps.

In the original work by Petry et al.^3^, some features were used with a cyclic-feature encoding (sine and cosine). In order to facilitate the interpretation of the LRP relevance results, we changed the encoding of these features to one-hot encoding and trained a model with this new input representation. As an additional preprocessing step we normalized numerical features to mean zero and unit variance. Further details on the data collection can be found in the prior work of Petry et al.^3^.

**Table 1:** Input features for the pollution forecasting model, including their source and unit/representation.

Input feature	Source	Unit
NO	LANUV^a^	μg/m^3^
NO$_{2}$	LANUV/LUBW^b^	μg/m^3^
O$_{3}$	LANUV	μg/m^3^
PM$_{10}$	LANUV	μg/m^3^
Air temperature	DWD^c^	°C
Precipitation	DWD	mm
Sunshine	DWD	%
Humidity	DWD	%
Wind direction	DWD	One-hot encoding (16 directions)
Wind speed	DWD	Beaufort scale (integer)
Year	Preprocessing	Integer
Month	Preprocessing	One-hot encoding (12 dimensions)
Weekday	Preprocessing	One-hot encoding (7 dimensions)
Hour	Preprocessing	One-hot encoding (24 dimensions)
Work-free day	Preprocessing	One-hot encoding (2 dimensions)
Station number	Preprocessing	Integer

^a^https://www.lanuv.nrw.de/landesamt/daten-und-informationsdienste.

^b^https://udo.lubw.baden-wuerttemberg.de.

^c^https://www.dwd.de/EN/ourservices/opendata/opendata.html.

### Sequence-to-sequence model

**Figure 1:**
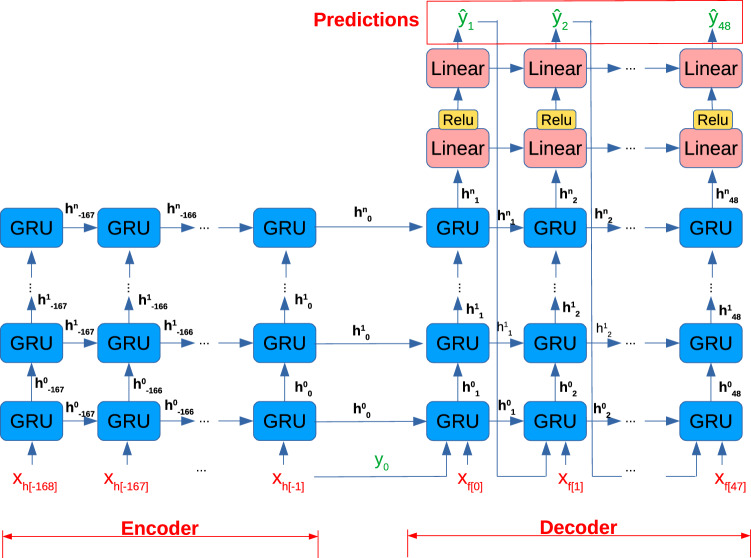
Sequence-to-sequence encoder-decoder neural network model, as introduced in Petry et al.^3^.

The sequence-to-sequence pollution forecasting model we use in the present work is similar to the one introduced in Petry et al.^3^. It contains two recurrent neural network (RNN) modules with each two Gated Recurrent Units (GRU) layers of dimension 1024. The decoder module additionally contains a fully-connected linear layer of size 1024 followed by a ReLU activation, plus a linear output layer without bias. Discarding the bias from the output layer is important for the XAI decomposition, such that when explaining the model’s predictions we are able to decompose the *full* output value into input variables’ contributions (indeed most XAI methods, including LRP, ignore the output layer’s bias in the relevance decomposition process). The output layer has size four, and we train one model to predict all four pollutants ($PM_{10}$, *NO*, $\text{NO}_2$ and $\text{O}_3$) at once. The resulting model is depicted in Fig. 1. The model is trained by minimizing the mean squared error, using the AdamW optimizer and a batch size of 128. The trained model has a mean absolute prediction error of 3.73 μg/m^3^ on the validation set.

Data is fed into the model sequentially 1 h at a time. The encoder receives as input the data of the last 7 days (168 h), which is called *historical* input (we refer to this data via the subscript *h* in the features). In each time step of the prediction, the decoder receives as input the weather forecast of the current hour, together with the pollution forecast of the last hour, plus the temporal input features and the static input, and makes a new prediction. The input data of the decoder module is called *forecast* input (we refer to this data using the subscript *f* in the features, or using the keyword *intermediate* in the case of the pollutant forecasts in prior time steps).

The model provides a forecast of the pollutants particulate matter ($PM_{10}$), nitrogen monoxide (*NO*), nitrogen dioxide ($\text{NO}_{2}$) and ozone ($\text{O}_{3}$) for the next 2 days (48 h). The model’s predictions are denoted by $\hat{y}$ in Fig. 1 (as opposed to *y* which denotes the true value of the pollutants). More information on the implementation details can be found in Petry et al.^3^.

## Explaining predictions

### Layer-wise relevance propagation (LRP)

LRP is a method to explain the decisions of a neural network model by computing the contribution of each input feature towards the predicted output^7^. More precisely, LRP redistributes layer-by-layer relevance scores from the output layer until the input layer neurons using a layer-wise relevance conservation property as shown in Eq. (1). The sum of the scores that neurons of the lower-layer receive from the upper-layer is conserved across all layers^7,12^ (up to the relevance which will be absorbed by the hidden layer “bias neurons” which we depict through the index 0 in Eq. 1).

1 $$\begin{aligned} f(\textbf{x})= R_{1}^{L} =...=\sum _{d=0}^{N_{l+1}}R_{d}^{(l+1)}= \sum _{d=0}^{N_l}R_{d}^{(l)}=...= \sum _{d=0}^{N_0}R_{d}^{(0)}, \end{aligned}$$

where $f(\textbf{x})$ is the model’s prediction for one target output neuron of interest (in our case the prediction for one pollutant type), $\textbf{x}$ is the model’s input, $R_{d}^{(l)}$ is the relevance of neuron *d* in layer *l*, and $N_l$ is the number of neurons (i.e., the dimension) of layer *l*. Further, we denote by $R_{1}^{L}$ the relevance of the output neuron of interest, *L* being the final layer of the model, which is set equal to $f(\textbf{x})$ for initializing the LRP backward propagation process.

Hence, every neuron in the network will get assigned its own relevance score, up to the input layer neurons which represent the input features’ contributions. The backward relevance propagation process of LRP is implemented in practice by specific LRP propagation rules which depend on the type of the network layer involved in the process. These rules have been theoretically justified via the mathematical framework of Deep Taylor Decomposition^53,54^. In the next subsection we will provide more details on these rules.

### LRP for a Seq-2-Seq model with GRUs

In this work we extend LRP to a sequence-to-sequence model with GRU layers. Given an input sequence indexed by *t*, the GRU cell^31–33^ performs the following recurrence computations, as provided in Eq. (2):

2 $$\begin{aligned} r_t & =\sigma (W_{ir} x_t+b_{ir} + W_{hr} h_{(t-1)}+b_{hr}) \\ z_t & =\sigma (W_{iz} x_t+b_{iz} + W_{hz} h_{(t-1)}+b_{hz}) \\ n_t &=\tanh (W_{in} x_t+b_{in} + r_t \odot (W_{hn} h_{(t-1)} +b_{hn}))\\ h_t &=(1-z_t)\odot n_t + z_t \odot h_{(t-1)} \end{aligned}$$

where $h_t$ and $x_t$ are the hidden state and the input vectors at time step *t*, and $h_{(t-1)}$ is the hidden state from the previous time step. $r_t$ and $z_t$ are the reset and update gates, and $n_t$ is the new candidate hidden state. ($W_{ir}$ $\vert$ $W_{iz}$ $\vert$ $W_{in}$) and ($b_{ir}$ $\vert$ $b_{iz}$ $\vert$ $b_{in}$) are input-to-hidden weights and biases. ($W_{hr}$ $\vert$ $W_{hz}$ $\vert$ $W_{hn}$) and ($b_{hr}$ $\vert$ $b_{hz}$ $\vert$ $b_{hn}$) are hidden-to-hidden weights and biases. $\sigma$ is the sigmoid activation function and is used for gate neurons, while $\tanh$ is the tanh activation, and both non-linear activations are applied element-wise. $\odot$ denotes an element-wise multiplication. The remaining operations are standard vector addition and matrix-vector product. All lowercase variables are vectors, and uppercase variables are matrices.

Hence a GRU cell consists of the three following types of layers, and for each of them we describe below the LRP rule that we employ to redistribute the relevance scores from the upper-layer neurons onto the lower-layer neurons:

- *Linear layer*. For a linear layer with $z_i$ representing the lower-layer neurons, weights $w_{ij}$ and biases $b_j$, in the forward pass the upper-layer neurons $z_j$ are computed using $z_j=\sum _i z_i\cdot w_{ij} +b_j$. To compute relevance scores of the lower-layer neurons $R_i$, given the relevances of the upper-layer neurons $R_j$, we follow the LRP-*epsilon* rule (LRP-$\epsilon$)^7,58^:
  3 $$\begin{aligned} R_{i\leftarrow j}= \frac{z_i\cdot w_{ij}}{z_j + \varepsilon \cdot sign(z_j)} \cdot R_j \end{aligned}$$
  where $R_{i \leftarrow j}$ is the share of relevance that neuron *i* receives from neuron *j* and $\varepsilon$ is a small positive number that is used as a numerical stabilizer. The relevance $R_i$ is finally computed as $R_i = \sum _j R_{i\leftarrow j}$, i.e. the sum of all relevance shares that a neuron receives from the upper-layer neurons connected to it.
- *Multiplicative layer*. Another type of layer occuring in a GRU cell is multiplicative interactions where the value of the upper-layer neuron in the forward pass is computed as the product of two lower-layer neurons, which we call gate and signal neurons, where the gate is the neuron that is sigmoid activated. For this type of layer, we utilize the LRP ”signal-take-all” redistribution rule (LRP-*all*)^13,58^, which redistributes all the relevance from the product to the signal neuron. For instance, in Eq. (2) last line, neurons in the hidden state $h_{(t-1)}$ will get assigned all the relevance from the product term $z_t \odot h_{(t-1)}$.
- *Activation layer*. In the case of element-wise non-linear activation layer such as *tanh* and *sigmoid*, the relevance score from the upper-layer would be transferred to the lower-layer without any changes (identity redistribution). Note that this doesn’t imply that the activation function is ignored by the LRP backward propagation process. In fact the *activated* neuron’s value is taken into account through the LRP relevance redistribution in the next higher linear layer.

Further, as mentioned earlier, in order to initialize the LRP backward propagation process, the relevance score of the output neuron of interest, i.e. for the pollutant prediction we want to explain, is set to the neuron’s predicted value, i.e. to the pollutant’s concentration for the considered prediction time step, while the relevances of the other output neurons are set to zero. This predicted quantity will be redistributed layer-by-layer through LRP up until the input neurons, i.e. in our case onto the historical input features, as well as the forecast input features from previous time steps plus the current time step, according to the sequence-to-sequence model’s architecture.

In order to check the correctness of our LRP implementation we performed a relevance conservation sanity check, where we verifed that the sum of the input neurons’ relevances is equal to the model’s predicted output for the pollutant and time step of interest. For this particular sanity check setup we redistributed the relevance assigned to the hidden layer biases uniformly onto lower-layer neurons (as per default in the standard LRP setup a share of relevance is absorbed by the hidden layer biases, and thus relevance is not exactly conserved).

For more details on our LRP implementation we refer to our released code (https://github.com/Sara-mibo/LRP_EncoderDecoder_GRU).

### Validating LRP through a toy task

In a previous work the authors designed a toy task to evaluate and compare XAI methods on recurrent networks^13^. Inspired by this approach, we design an arithmetic toy task to verify the LRP relevance scores objectively on our sequence-to-sequence model. As the relationship between inputs and outputs is known, we can use this task to validate the correctness of the LRP relevance results. These relevances should represent the contributions of the input features to the output accordingly to our data generation process.

**Figure 2:**
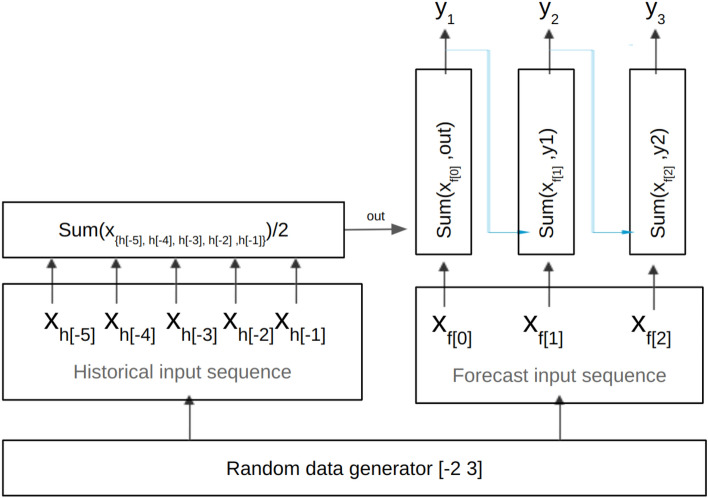
Toy task generation process. Labels *y* are generated via simple sum and division on the sequence of two-dimensional inputs of random numbers sampled in the range [− 2 3].

To perform this experiment, we use a sequence-to-sequence model similar to the pollution forecasting one, but with fewer input features (we use only two input features) and a hidden layer dimension of 128. The data generation process is shown in Fig. 2. Output labels are created by performing simple operations such as sum and division on the sequence of random input features. 100,000 data points are generated with sequence lengths of five for historical inputs and three for forecast inputs, and split into train and validation sets in a ratio of four to one.

Same as with the pollution forecasting model, we have an encoder and a decoder module, each fed with a sequence of inputs, either historical or forecast inputs. According to the data generation process depicted in Fig. 2, to generate an output label *y* all the inputs from the historical input sequence are summed up and divided by two. The result is then passed to the decoder module. In each prediction time step for generating labels, the forecast input and the output from the previous time step are added together. If the model learned the input-output relationship correctly, which we ensured via model training, then LRP is supposed to compute the relevances in such a way that every element in the historical input contributes to the prediction with half of its value, while the contribution of each element in the forecast input, up to the current time step, should be equal to its value.

**Figure 3:**
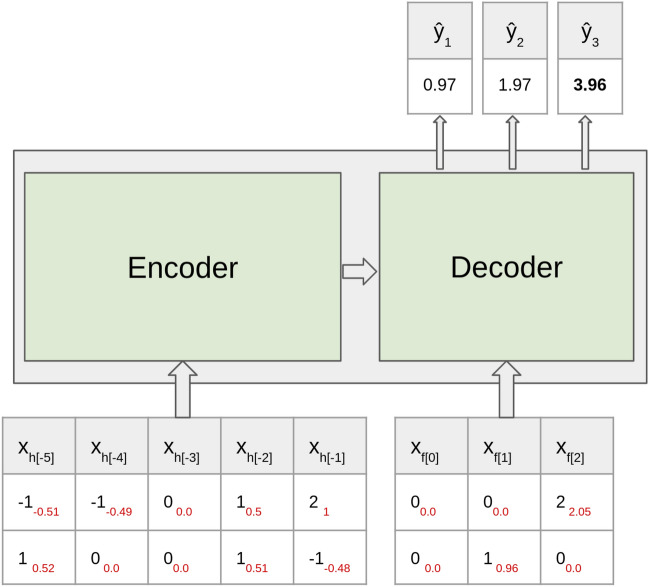
Toy task LRP relevance results. We explain the last time step of the prediction (marked in bold). The relevance score of each input is shown in red, beside the input’s value in black.

Given an input example, the true labels *y* and the predictions $\hat{y}$ of the trained model are shown in the box below:
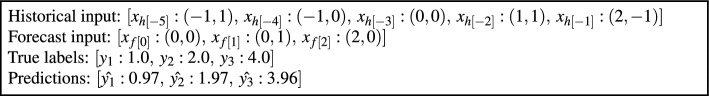


On the example above, we perform an LRP decomposition on the last time step of the predicted output sequence, i.e. we explain the prediction $\hat{y_3}$. The corresponding relevance scores of the inputs computed with LRP are shown in red, beside the value of the inputs in black, in Fig. 3.

From Fig. 3 we observe that the contributions of historical inputs are almost half of their values, and the relevance scores of forecast inputs are close to their value. Negative inputs get assigned negative contributions to the output due to their subtractive role in the summation. In other words, we can recover the input sequences from the LRP relevances by multiplying relevances of the historical inputs by two and keep the relevances of the forecast inputs unmodified. Thus LRP redistributes relevance scores among inputs as expected.

## Explaining pollution forecasts

Our sequence-to-sequence model forecasts the concentration of four major pollutants, namely $PM_{10}$, *NO*, $\text{NO}_2$ and $\text{O}_3$ for the next 48 h, as was described in the “Sequence-to-sequence model” section. Now using LRP as an explanation method, we analyse the contributions of the input features to high pollutant concentrations, and to this end we begin by performing some statistics on the LRP relevance scores.

For each type of pollutant, we filter 4800 data points with the highest predictions in the first day of the forecast (more precisely for each hour of the first 24 h of the forecast 200 points are selected). For these data points, we compute the LRP relevance scores by explaining the given predictions (for features that are one-hot encoded, we obtain the relevance of the feature by simply summing up the relevances across the input vector dimensions, which is equal to the relevance of the non-zero input vector entry). Then, we compute the average of the positive relevance scores for every hour of the first day of the forecast, and additionally, for a few specific input features’ values we compute the average of the positive relevance scores *across* different hours of the first day of the forecast (in particular for the temporal input features hour of the day, day of the week, month of the year, working day vs. work-free day).

The resulting statistics over input features can be visualized as two-dimensional heatmaps (the more relevant the feature, the deeper the color), where the x-axis represents the prediction hours (i.e., the forecast time steps) and the y-axis represents the input features, as well as, for specific input features where the average was performed *across* prediction hours, as bar plots over the possible input feature’s values (where the height of the bar represents the relevance of that feature’s value). Both visualizations help us to quickly identify features with the highest positive contributions towards high pollution predictions, as well as determine the relative importance of some specific features’ values over time (hours, days, months) for high predictions.

However, an important point to note here is that these heatmaps represent the *contributions* of the input features to the pollution forecast, but do not indicate the corresponding input features’ *values*, i.e. their magnitude and range. For instance, we can not determine from the LRP heatmap whether the low temperature is positively contributing to high pollution, or whether it’s high temperature. Therefore we need to average the inputs too in order to determine which input values are positively contributing to high pollution forecasts. Hence we compute the average of the input values on the subset of data points that was previously selected as discussed above, as well as the mean and standard deviation of these features over the whole dataset, and report them in Table 2. For more brevity only the statistics of the most relevant features per pollutant (as identified through the heatmaps, see subsequent sections) are retrieved in Table 2.

**Table 2:** Input features with the highest positive LRP relevance for high predictions of each pollutant (4800 data points with high predictions were selected considering the first 24 h of the forecast).

Input feature	PM$_{10}$	NO	NO$_{2}$	O$_{3}$	Mean (all)	Std (all)
Temperature_f (${}^\circ$C)	− 0.8 (6)	2.5 (5)	15 (9.6)	26.3 (5.8)	10.5	7.4
Humidity_f (%)		85 (13.5)	65 (21)	57 (20.9)	77.5	16.8
Hour_f (one-hot)		7, 8, 9			–	–
WindSpeed_f (Bft)		2 (1.3)	2 (1.1)		3.6	2
PM10_h (μg/m^3^)	89.5 (33)				18.6	10.5

Columns 2 to 5 are the average input features’ values on the considered subset of data points (and standard deviation in parenthesis), while the last two columns contain the mean and standard deviation of these features over the whole dataset. Subscript *f* indicates forecast input, subscript *h* indicates historical input. For the one-hot encoded input feature hour of the day the hours 7, 8, 9 A.M. are the 3 h that occur the most in the subset of high predictions (over the whole dataset all hours occur equally often).

**Figure 4:**
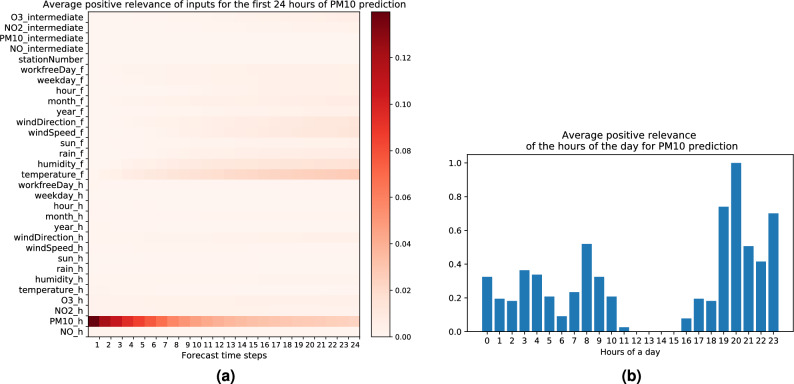
(**a**) Average positive LRP relevance for the first 24 h of high $PM_{10}$ forecasts. Subscript *h* and *f* are used for historical and forecast input. Subscript *intermediate* is used for pollutant forecasts in previous time steps. Station number is the static input. (**b**) Average positive LRP relevance for the input feature hour of the day for high $PM_{10}$ forecasts (for the bar plot quantities are rescaled such that the maximum is equal to one). Statistics are computed over 4800 data points with the highest $PM_{10}$ forecasts [created with Matplotlib version 3.3.4 https://matplotlib.org/].

**Figure 5:**
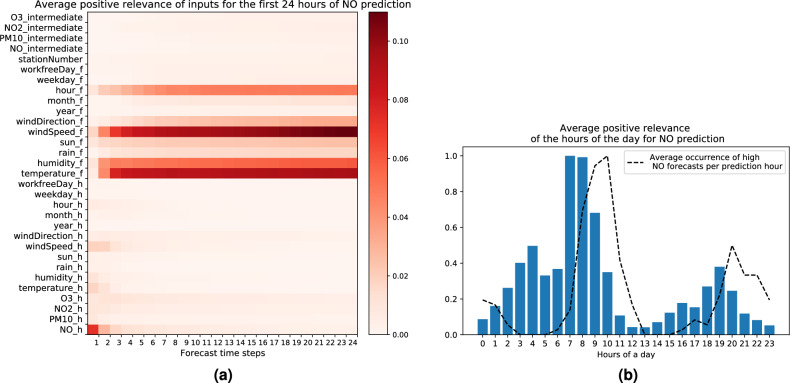
(**a**) Average positive LRP relevance for the first 24 h of high *NO* forecasts. Subscript *h* and *f* are used for historical and forecast input. Subscript *intermediate* is used for pollutant forecasts in previous time steps. Station number is the static input. (**b**) Average positive LRP relevance for the input feature hour of the day for high *NO* forecasts (for the bar plot quantities are rescaled such that the maximum is equal to one). Additionally we plot the average occurrence of high *NO* forecasts per hour of the prediction time (dotted line). Statistics are computed over 4800 data points with the highest *NO* forecasts [created with Matplotlib version 3.3.4 https://matplotlib.org/].

**Figure 6:**
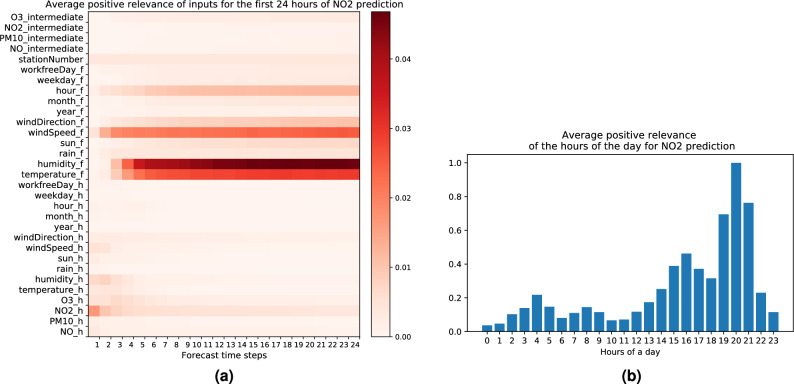
(**a**) Average positive LRP relevance for the first 24 h of high $\text{NO}_2$ forecasts. Subscript *h* and *f* are used for historical and forecast input. Subscript *intermediate* is used for pollutant forecasts in previous time steps. Station number is the static input. (**b**) Average positive LRP relevance for the input feature hour of the day for high $\text{NO}_2$ forecasts (for the bar plot quantities are rescaled such that the maximum is equal to one). Statistics are computed over 4800 data points with the highest $\text{NO}_2$ forecasts [created with Matplotlib version 3.3.4 https://matplotlib.org/].

**Figure 7:**
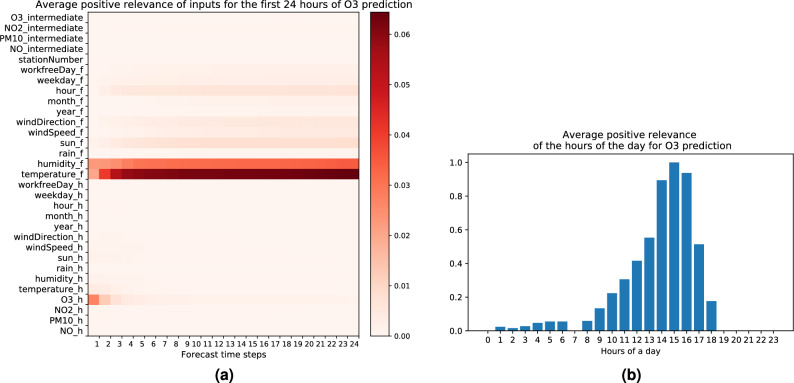
(**a**) Average positive LRP relevance for the first 24 h of high $\text{O}_3$ forecasts. Subscript *h* and *f* are used for historical and forecast input. Subscript *intermediate* is used for pollutant forecasts in previous time steps. Station number is the static input. (**b**) Average positive LRP relevance for the input feature hour of the day for high $\text{O}_3$ forecasts (for the bar plot quantities are rescaled such that the maximum is equal to one). Statistics are computed over 4800 data points with the highest $\text{O}_3$ forecasts [created with Matplotlib version 3.3.4 https://matplotlib.org/].

In the following we will interpret our LRP results regarding the most relevant input features per pollutant, as well as the distribution of relevance for specific input features’ values for high pollution forecasts, and we contrast our findings with domain knowledge from prior works in order to assess the pertinence of an eXplainable AI method such as LRP in the environmental domain. Note that when interpreting the heatmaps (Figs. 4a, 5a, 6a, 7a) we always implicitly back up our findings upon Table 2 to identify which values (high or low) of the input features led to high predictions (by comparing their average on the subset of high prediction data points to their mean value over the whole dataset).

### Explaining high PM_10_ forecasts

In Fig. 4a we plot the average of the positive LRP relevance for high forecasts of the pollutant $PM_{10}$. We find that the following input features are the most relevant ones for high levels of $PM_{10}$ concentration in the air:

- *High* $PM_{10}$ *measurement.* We find that high values of $PM_{10}$ measurements in the historical input sequence have the strongest impact on the forecast of high $PM_{10}$ concentrations, especially in forecasts for the next hour, this effect then declines over time. $PM_{10}$ particles are usually produced from industrial and combustion processes, and through fugitive dust emission. Coarse particles of $PM_{10}$ are known to easily deposit, and typically travel less than ten km from their place of generation^63^. Accordingly $PM_{10}$ particles that are produced nearby and detected through sensor measurements can be mainly responsible for the amount of $PM_{10}$ concentration predicted in the next hours. We find that the locations of stations with high $PM_{10}$ concentrations are indeed close to industrial plants such as cement, steel or iron manufacturing plants, along with fossil fuel power plants. For example station DMD2, which is located in the city of Dortmund, has several industrial firms closely situated to it, like the Holcim HüttenZement cement fabricator, the DGW energy plant, and the Thyssenkrupp steel fabricator. Such industries are known to emit a significant amount of particulate matter into the air^64^.
- *Low temperature forecast.* A low temperature forecast also contributes to a high $PM_{10}$ load in the air. The phenomenon of temperature inversion might explain this result. Indeed temperature inversion in cold days of the year is known to bring additional accumulation of pollutants in the atmosphere close to the ground^65^. Temperature inversion happens when the warm air floats above the cooler air near the surface. This warm layer of air can play the role of a lid and trap the pollution close to the ground. Besides, the high pollutant concentration during cold days can also be a consequence of additional emissions due to domestic heating, e.g. through fossil fuel combustion^66^.

In Fig. 4b, the contributions of different hours of the day to the high $PM_{10}$ concentrations are shown. Even though the hour input feature does not have a high relevance score overall for the prediction of $PM_{10}$, compared to the historical $PM_{10}$ measurement and the forecasted temperature (as seen in heatmap Fig. 4a), we can still determine which hours of the day are more relevant than others, relatively. As it can be observed from the bar plot, hour 8 P.M. contributes the most to the pollution load, and the evening hours until midnight tend to have a higher contribution than late night hours after midnight (and before 8 A.M.). This could be attributed to the higher domestic heating needs in the evening after sunset, in comparison to daytime and throughout the night when people are sleeping^65^.

### Explaining high $\textbf{NO}$ forecasts

Similar to the experiment with $PM_{10}$, we plot the average of the positive LRP relevance for high forecasts of *NO* in Fig. 5a. We find that the following input features contribute most towards high *NO* concentration:

- *Low wind speed forecast.* Wind usually plays a role in reducing the pollution load in an area by dispersing the pollutants. Conversely an absence of wind allows for pollutants to pile up. As a result, low wind speed becomes a contributor to an increase in pollution load. This could explain the high relevance scores we observe for low wind values with respect to high *NO* predictions, accordingly to Fig. 5a and Table 2. This result is also consistent with previous studies that found a strong negative correlation between *NO* concentration and wind speed^67–69^.
- *Low temperature forecast.* As we observed with the pollutant $PM_{10}$, we find that low temperature contributes to the prediction of high *NO* concentration. Again the reason could be the temperature inversion phenomenon or the significant increase in burning fossil fuels for heating and electricity generation during cold days^70,71^.
- *High humidity forecast.* The heatmap in Fig. 5a also suggests a positive contribution of high humidity to high *NO* concentration. *NO* generated by natural or anthropogenic sources is a reactive gas and rapidly oxidizes to $\text{NO}_2$, in particular through reaction with ozone. Due to this reaction large concentration of ozone and *NO* can not coexist^72^. Since high humidity is generally associated with lower solar radiation and less ozone formation, we hypothesise this could explain why fewer nitrogen monoxide is converted into nitrogen dioxide when the relative humidity is elevated, allowing *NO* to reach higher concentration. In the previous literature however we could not find such a relationship between high *NO* pollution load and high relative humidity. Thus our interpretation of this result shall be taken with caution, and there might be other mechanisms at play that better justify the high humidity contribution to the model’s prediction.
- *Morning hours in the forecast.* Finally Fig. 5a shows high relevance scores for the hour of the day feature in the forecast input sequence. Additionally, Fig. 5b represents the average contribution of each hour of the day to high *NO* predictions across various forecast time steps. The hours of the day that positively contribute the most to the *NO* load in the air are around 7 A.M.–8 A.M. in the morning. This is consistent with the diurnal variations of pollutants found in previous work^8^, presenting a major peak of *NO* concentration that starts early in the morning and lasts till late morning. Generally, high emissions of $NO_x$ coincide with rush-hour traffic occurring in the morning and late afternoon. However, in the late afternoon, the level of ozone concentration increases because of the presence of solar radiation during the day that promotes ozone generation. As a result, due to the oxidation of *NO* to $\text{NO}_2$ through the chemical reaction previously mentioned of $\text{O}_3 + \text{NO} \, \longrightarrow \, \text{NO}_2 + \text{O}_2$, the concentration of *NO* in the late afternoon is not as high as it is in the morning^73^. In Fig. 5b, beside the average contribution of each hour input feature to high forecasts (as a bar plot), we additionally plot the average occurrence of high *NO* forecasts for each prediction time step (as a dotted line). We observe that the distribution of high forecasts of *NO* approximately follows the same pattern as the contribution of the input feature hour of the day, but with a slight delay of around 2 h. This is probably due to the accumulation effect of pollution, meaning that *NO* produced at relevant input hours accumulates into the air to increase the *NO* prediction at subsequent hours.

### Explaining high NO_2_ forecasts

Figure 6a depicts the average positive LRP relevance for high forecasts of $\text{NO}_2$. We find the following input features to contribute substantially towards high levels of $\text{NO}_2$:

- *Low humidity forecast.* Nitrogen dioxide $\text{NO}_2$ is an acidic gas and readily reacts with water vapor to generate nitric acid and nitrogen monoxide through the reaction of $3 \text{NO}_2 \, + \, \text{H}_2\text{O} \, \longrightarrow \, 2 \, \text{HNO}_3 \, + \, \text{NO}$. This is also the reaction that causes acid rain formation from nitrogen dioxide present in the atmosphere. Therefore, when humidity is high $\text{NO}_2$ can be consumed and removed from the air through nitric acid deposition, and conversely, when the humidity is low this could contribute to $\text{NO}_2$ accumulation^74^.
- *Moderately high temperature forecast.* As can be seen from Fig. 6a and Table 2, an average temperature of 15 °C (with standard deviation of 9.6 °C) seems to act in favor of a high $\text{NO}_2$ concentration. We suspect this temperature level to not be low enough to explain higher emissions due to fossil fuels combustion for heating or accumulation of pollutant through temperature inversion, and at the same time to not be high enough to justify a high ozone concentration which could lead to a secondary formation of $\text{NO}_2$ from oxidation of *NO*. Previous work found that indeed the $\text{NO}_2$ concentration is rather affected by temperature levels either lower than 5 °C or higher than 20 °C^75^. Therefore it is possible that other mechanisms are at play here to increase $\text{NO}_2$ formation, e.g. via nitrogen oxides emissions from micro-organisms present in the soil^76^. More investigations would be needed to understand the reasons behind the role of temperature in this context, as well as possible interactions with other factors not modelized in our approach.
- *Low wind speed forecast.* Similarly to what we observed with the pollutant *NO*, the low wind speed also allows $\text{NO}_2$ to accumulate since the dispersion and transport of pollution is hindered, as opposed to when the wind speed is high.

According to the bar plot in Fig. 6b, the time of the day around 8 P.M. contributes the most to high $\text{NO}_2$ forecasts, and late afternoon hours contribute more than morning hours. This could be attributed to the contributions from evening rush-hour traffic, emissions due to heating, and the secondary formation of $\text{NO}_2$ from the reaction between primary emitted *NO* and $\text{O}_3$^77^.

### Explaining high O_3_ forecasts

Figure 7a depicts the average of the positive relevance of the input features for high forecasts of $\text{O}_3$. The most important input features for predicting high $\text{O}_3$ concentration are the following:

- *High temperature forecast.* Elevated temperature displays a positive contribution to the ozone pollution load. The reason is that higher temperature is representative of more solar radiation, which supports the photo-chemical formation of $\text{O}_3$. Indeed solar energy is known to play a key role in ozone formation (see e.g.^9,10,78,79^).
- *Low humidity forecast.* As already mentioned, ground-level tropospheric ozone is formed as a result of complex photo-chemical reactions^9,10,79^. The corresponding cyclic reactions involve $NO_x$, Volatile Organic Compounds (VOCs) and *CO*. High humidity is generally associated with cloudy sky and precipitation, resulting in weaker solar radiation, while on the other hand low humidity favors solar radiation which enhances ozone production^80^. This explains in great part the high contribution of a low humidity forecast on ozone concentration observed in Fig. 7a. Another complementary explanation for this result might be dry deposition, i.e. the uptake of ozone by trees^11^. Indeed when the relative humidity is high trees open their stomata for exchanging $\text{CO}_2$, and then unintentionally also absorb $\text{O}_3$ from the air, while when the humidity is low trees close their stomata such that they don’t dry out, and consequently they do not remove $\text{O}_3$ from the air, allowing it to pile up.

In Fig. 7b we visualize the average contribution of the input feature hour of the day for high $\text{O}_3$ forecasts. We observe that the most relevant hours are during daytime, and especially between noon and 5 P.M. This is consistent with the diurnal cycle of solar radiation and its impact on $\text{O}_3$ concentration, with a maximum concentration occuring around 3 P.M. (i.e. approx. 2 h after the maximum of solar radiation) as was found in previous work^78^.

Further statistics on the relevance for other temporal input features like months of the year, days of the week and work-free versus working day for high pollution forecasts of the different pollutants can be found in the Supplementary Section 1.

### Influence of wind direction

**Figure 8:**
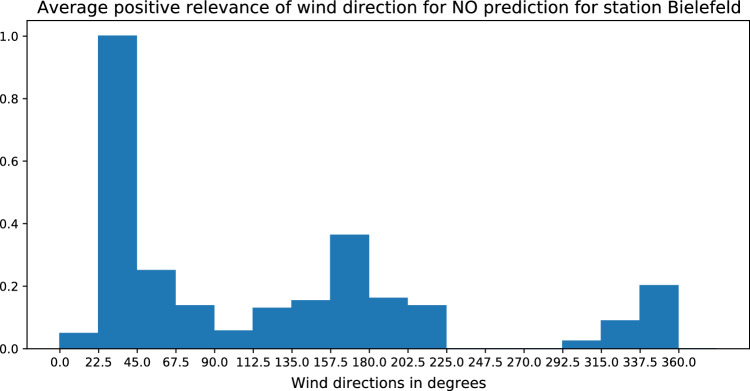
Average positive LRP relevance for the input feature wind direction for high NO forecasts for station BIEL in Bielefeld. This statistic is computed over 71 data points with the highest forecasts of NO.

**Figure 9:**
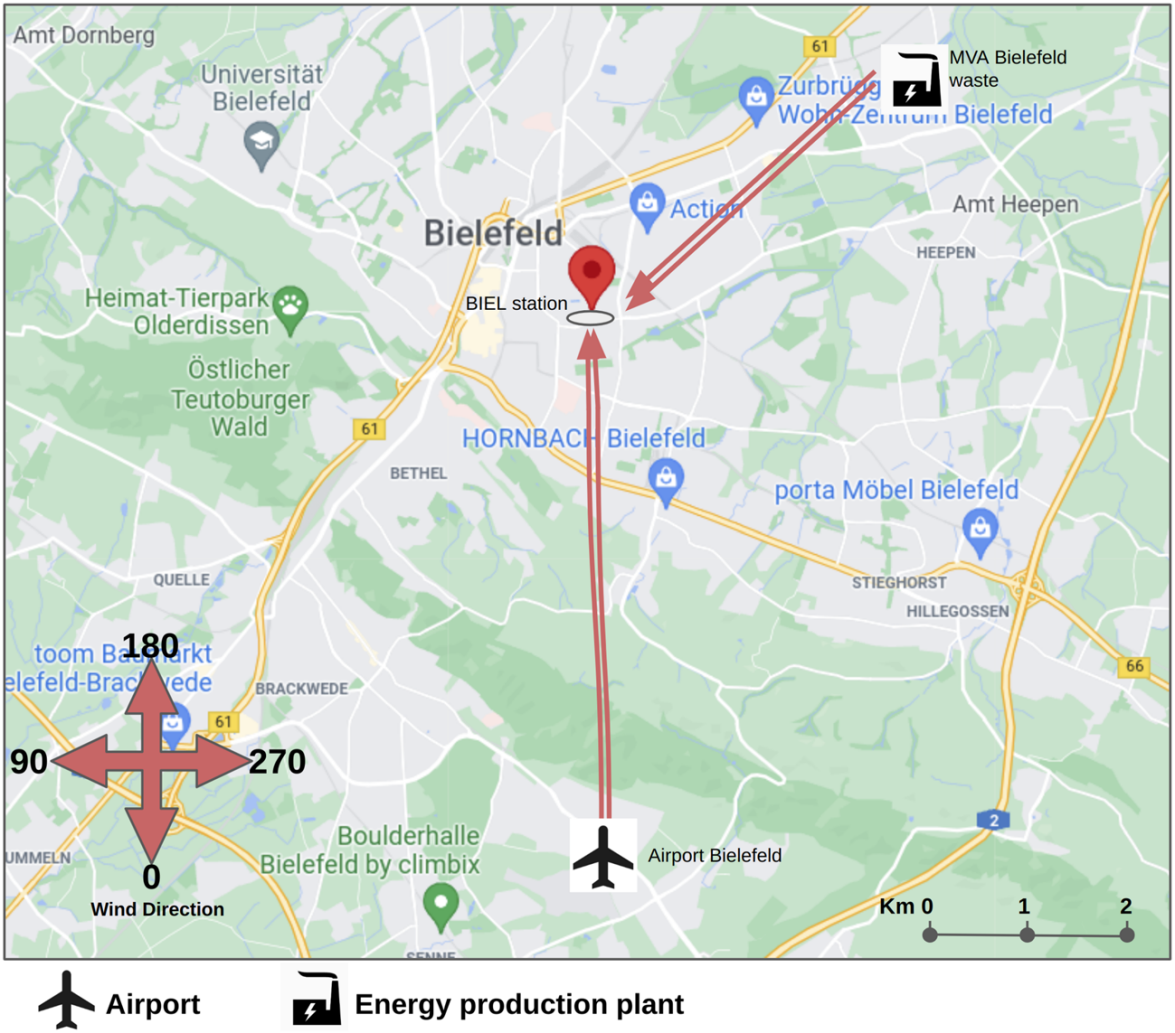
Locations of the station BIEL in Bielefeld and potential pollution sources, as well as relevant wind directions identified via LRP [created with Google Maps https://www.google.com/maps and Google Slides https://www.google.com/slides/about].

In the previous subsections we analysed the relevance of various input variables on the forecast of high pollutant loads. In this subsection we investigate in more details a particular input feature which contributes to high forecasts for the pollutants *NO* and $\text{NO}_2$: the wind direction. To determine the most important wind directions, the relevance of the different wind directions is computed for one specific station. In particular we consider the station BIEL in Bielefeld which has high forecasts of *NO*. We average the positive LRP relevance of the input feature wind direction for that specific station across different hours of the prediction time with high forecast values. This statistic is computed over 71 data points with the highest forecasts of NO. The result is shown in Fig. 8. According to the bar plot, wind that blows in the direction between 22.5°–45° enhances pollution the most, while the next peak can be found in the range 157.5°–180°.

The location of the measuring station and the two most influential wind directions are depicted in a map in Fig. 9. As reported by the European Environment Agency^81^, energy production, as well as road and non-road transport generate a large share of *NO* emissions. In accordance with that we find two sites that could be responsible for the high emissions and which are consistent with the relevant wind directions identified via LRP: the Bielefeld airport and an energy production plant. This illustrates the usefulness of LRP for identifying potential relevant pollution sources.

## Simplifying the model with LRP

**Figure 10:**
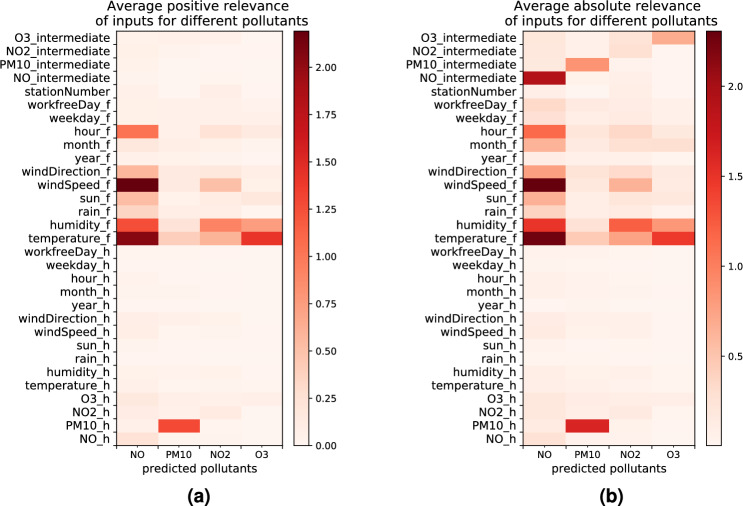
(**a**) Average positive LRP relevance for the first 24 h of high forecasts for different pollutants. (**b**) Average absolute LRP relevance for the first 24 h of high forecasts for different pollutants. Subscript *h* and *f* are used for historical and forecast input. Subscript *intermediate* is used for pollutant forecasts in previous time steps. Station number is the static input. Statistics are computed over 4800 data points with the highest forecasts for each pollutant [created with Matplotlib version 3.3.4 https://matplotlib.org/].

**Table 3:** Validation loss (as mean absolute prediction error) for two different models: the original encoder-decoder model and a decoder-only model.

Model	Loss (μg/m^3^)	Training time (*hours*)
Encoder–decoder	3.73	17.6
Decoder-only	4.63	7.5

**Figure 11:**
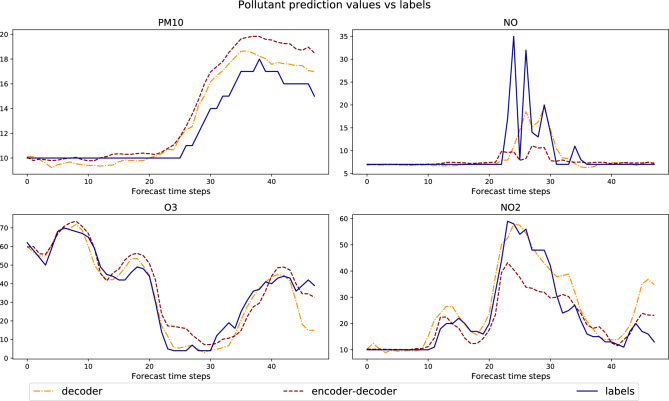
Pollution predictions by two different models: the original encoder-decoder model and a decoder-only model. *labels* are the true pollutant concentrations (in μg/m^3^).

According to the statistics on the positive, respectively the absolute, LRP relevance values computed over 19,200 data points with the highest forecasts for different pollutants in the first day of the prediction (4800 data points per pollutant) as shown in Fig. 10, we observe that the historical input data, except for the historical measurement of $PM_{10}$, do not contribute significantly to the pollution prediction. This indicates the model is mainly relying on the nearby forecast input data to make its prediction.

Considering this observation, we explored whether we could simplify the model’s structure by removing the encoder module from the model and re-train a new model with only the decoder module without loosing much prediction performance. In order to keep feeding the model with the last value of the historical measurement of $PM_{10}$, we included this value as input into the first time step of the decoder. The resulting validation loss and the training time for the modified and original models are given in Table 3. While the original encoder-decoder model receives a combination of historical and forecast inputs for training and prediction, and the decoder-only model just consists of the decoder fed with forecast data plus one measurement of $PM_{10}$, we find that both models reach reasonable prediction performance. Indeed there is only a slight increase in the validation loss when using the decoder-only model (in terms of mean absolute prediction error). We provide some example predictions on a few data points from the validation set in Fig. 11. This experiment demonstrates that explaining the model behavior with LRP can also be used to design a more efficient model. By omitting the encoder module, we finally obtain a model that needs less time and computational resources for training and prediction. The resulting model looses a bit of accuracy, but still solves the prediction problem with enough precision to fulfil practical expectations.

Similarly, we inspected the impact of removing a few irrelevant features as identified by LRP from the historical input sequence of the original encoder-decoder model, and verified whether this affects the prediction performance negatively, as was done in previous work^41^. For this analysis we discarded the following historical input features: year, weekday, work-free day, sunshine and precipitation (i.e. rain), which never were assigned a high relevance for the high pollutant forecasts (see relevance heatmaps in the previous section and in Fig. 10). In terms of validation loss for each pollutant, when we re-trained a new model with this modification, the resulting change in prediction performance was no more than 0.02 μg/m^3^, which is negligible. Hence the LRP relevance can not only help to understand and explain the key factors influencing the pollution forecast the most, but can also be used to improve model efficiency, and reduce the number of input features.

Lastly, we inspected the influence of the static input feature station number (which is represented as an integer indicating the location of the considered station, and can in principle be used by the model to adapt its predictions to the environmental specificities of a given location). According to the LRP heatmaps, this input feature also never was assigned a large relevance value for high pollutant forecasts. Therefore we would expect it can be discarded from the input data without much loss in prediction performance. And indeed when we re-train the original encoder-decoder model without that feature, we obtain a new model with an equivalent prediction performance (no more than 0.02 μg/m^3^ change in prediction performance per pollutant). This indicates that the remaining dynamic input features (such as weather forecast, previous pollution concentration and temporal features) contain enough information for the given pollution forecasting task at hand.

For a comparison of the LRP results with other XAI methods, we provide in the Supplementary Section 2 additional heatmap visualizations with the XAI methods of Saliency and Gradient x Input^46,47^.

## Conclusion

In this work we applied the LRP method on a trained sequence-to-sequence pollution forecasting model with GRU layers. The explanations produced by LRP helped us to better understand how the model makes its predictions. LRP heatmaps were qualitatively inspected, and their results were largely consistent with available domain knowledge. We expect LRP to be broadly useful for the fine-grained analysis of model behavior in the environmental domain, in particular for the identification of potential critical factors contributing to high pollution concentration, which could improve the air quality monitoring and mitigation. Further, we demonstrated that LRP can be used to design a computationally more efficient forecasting model. A possible future direction for investigation could be the identification of factors interacting together in the pollution forecast, e.g. through the successive removal of individual versus groups of variables, and their interpretation in terms of the involved chemical and physical processes. In this respect we believe that data driven and eXplainable AI could complement more traditional approaches for broadening the understanding of pollution mechanisms.

## Supplementary Information

Supplementary Information.

## Data Availability

The data is not publicly available. The data analysed in the study will be available from corresponding author on reasonable request. The code of the model is publicly available at https://github.com/Sara-mibo/LRP_EncoderDecoder_GRU.
