# Supplementary material for: Explainable sequence-to-sequence GRU neural network for pollution forecasting
Source: Sci Rep. 2023 Jun 19;13:9940. doi: 10.1038/s41598-023-35963-2 (PMC10279754; doi:10.1038/s41598-023-35963-2)
Supplement: Supplementary file 1 — Supplementary Information. [file 41598_2023_35963_MOESM1_ESM.pdf]

Explainable Sequence-to-Sequence GRU Neural Network for Pollution Forecasting
# – SUPPLEMENTARY INFORMATION –

Sara Mirzavand Borujeni[1], Leila Arras[1,3], Vignesh Srinivasan[1], and Wojciech Samek[1,2,3,*]

[1]Fraunhofer Heinrich Hertz Institute, Department of Artificial Intelligence, 10587 Berlin, Germany
[2]Technische Universität Berlin, Department of Electrical Engineering and Computer Science, 10587 Berlin, Germany
[3]BIFOLD – Berlin Institute for the Foundations of Learning and Data, 10587 Berlin, Germany
[*]wojciech.samek@hhi.fraunhofer.de

ABSTRACT

(see main part for the abstract)

1 Additional relevance results for temporal input features

Similarly to the Section "Explaining Pollution Forecasts", we provide statistics over the positive LRP relevance of input features for specific temporal features of interest. These statistics are computed over 4,800 data points with the highest forecasts per pollutant during the first 24 hours of the prediction. The results per input feature's value are finally visualized as a bar plot in order to get an estimate of the relative importance of these features over time towards high pollution predictions.

1.1 Relevance of the months of the year

Figure 1 represents the average positive LRP relevance of the months of the year for high forecasts of the different pollutants. According to the bar plots, warmer months of the year (July and August) contribute the most to high O_3 concentration, which is in line with domain knowledge indicating that solar radiation is a key ingredient for ozone formation (e.g.[1–4]). As for NO, the months close to the new year are the most relevant ones for high values of this pollutant. Indeed, during this period temperature is low and humidity is high in Germany. Hence, as already mentioned in the main part of this work, increased emissions, in particular through fossil fuels combustion for heating, greatly explain this result[5,6]. Finally, March and January present a high positive contribution to an increased concentration of both PM_{10} and NO_2. We couldn't find a well-established reason in the literature for supporting this result, thus we suspect this finding would warrant further investigation to be understood.

1.2 Relevance of the days of the week and working vs. work-free days

We now inspect the relevance for different days of the week, and for working days versus work-free days, according to Figures 2 and 3.

While all weekdays positively contribute to the amount of NO_x emissions in Figure 2, Mondays and Fridays are the days of the week that have the highest contribution for these pollutants. This could be derived from daily road traffic accompanied by weekend getaways. This stands in agreement with the higher relevance of workdays for high NO_x forecasts, as opposed to work-free days, as shown by Figure 3.

For the PM_{10} concentration, although the weekdays contribute more than weekends as shown by Figure 2, work-free days have a higher impact on the high pollution predictions, as can be seen in Figure 3. This means that holidays (days that could be weekdays and work-free at the same time) are decisive to high levels of the PM_{10} load. The reason could be special events, e.g. fireworks for the new year that release a huge amount of PM_{10} into the air. Reports from different cities around the world confirm that the events happening close to the new year can lead to extreme values of PM_{10} concentration[7,8].

For the forecast of high O_3 concentrations, according to Figures 2 and 3, weekends (Friday and Saturday) as well as work-free days are the most relevant days. A reason for this finding could be the phenomenon called Ozone Weekend Effect (OWE), which usually occurs in urban areas. This effect is characterized by a higher concentration of ozone during the weekends even though anthropogenic emissions of the O_3 precursors, such as VOCs and NO_x, are usually lower on weekends. This is because during the week the ozone level is dominated by the reaction $O_3 + NO \longrightarrow NO_2 + O_2$, which limits the

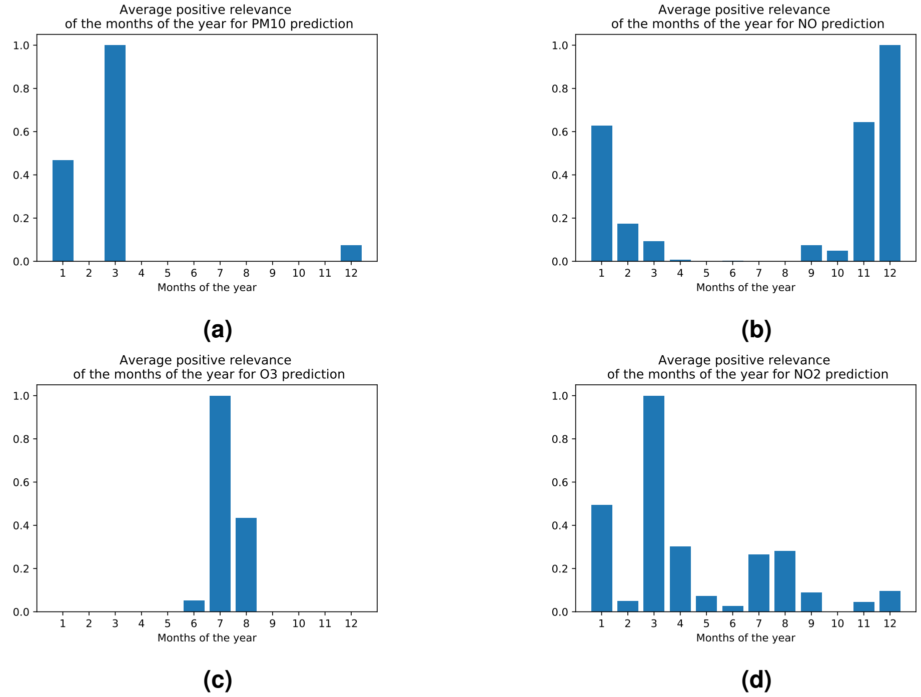

Figure 1. Average positive LRP relevance for the input feature months of the year for high forecasts of each pollutant (quantities are rescaled such that the maximum is equal to one). Statistics are computed over 4,800 data points with the highest forecasts per pollutant.

ground-level ozone concentration. However, during the weekend, as less NO is emitted from road transport, also less O_3 is consumed by the previous reaction, leading to a rise of O_3 concentration on weekends[9].

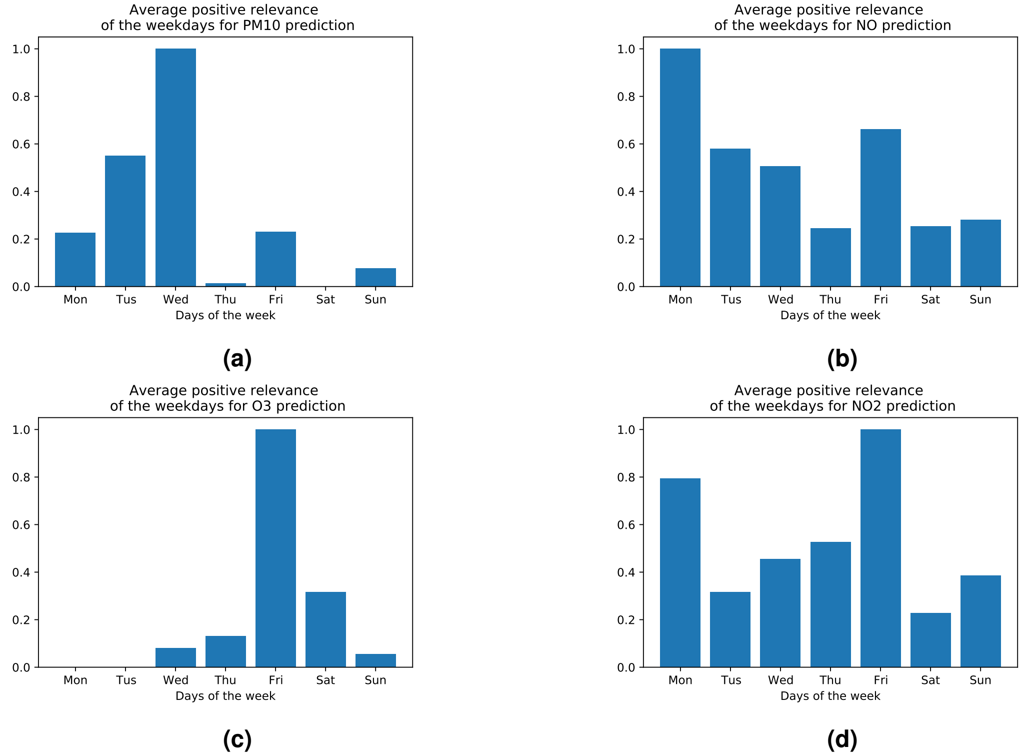

Figure 2. Average positive LRP relevance for the input feature days of the week for high forecasts of each pollutant (quantities are rescaled such that the maximum is equal to one). Statistics are computed over 4,800 data points with the highest forecasts per pollutant.

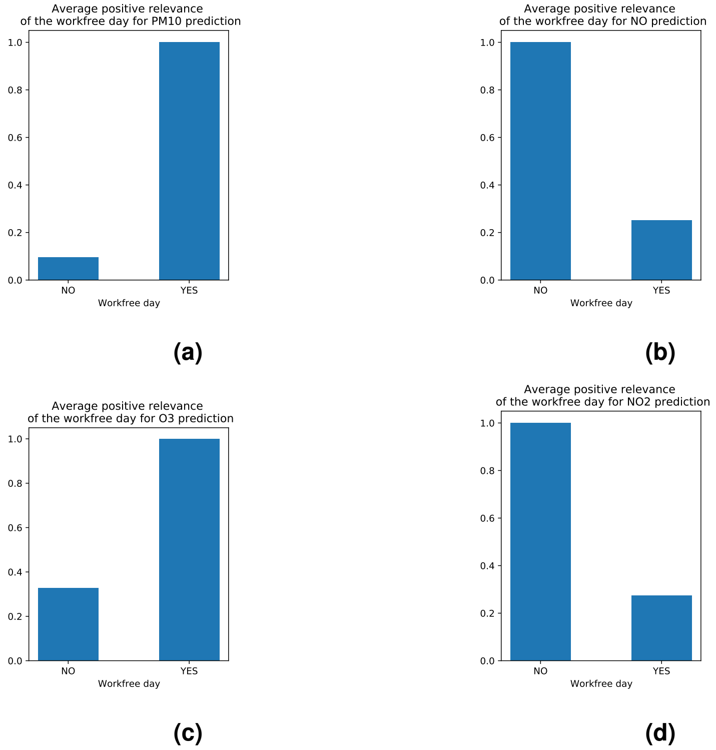

Figure 3. Average positive LRP relevance for the input feature working day vs. work-free day for high forecasts of each pollutant. Statistics are computed over 4,800 data points with the highest forecasts per pollutant.

2 Relevance results with other XAI methods

As already mentioned in the Subsection "Explanation Methods", we employ the XAI method of Layer-wise Relevance Propagation (LRP) in our experiments, as this method presents several advantages, in particular it can be computed in a single backward pass through the network and was shown to deliver superior results w.r.t. other XAI methods in various comparative studies (e.g.[10–13]).

Still, in order to get a sense of how the LRP results differ from other XAI methods, we provide in Figure 4 statistics of the positive, respectively the absolute, relevances computed over 19, 200 data points with the highest forecasts for different pollutants in the first day of the prediction (4,800 data points per pollutant). We consider the XAI methods of Saliency[14] and Gradient x Input[15], which we computed with the Captum toolbox[16] (version 0.4.1, for the Saliency method we set the `abs` argument to False). Note that these XAI methods are primarily intended to deliver relevances for the input features only, and not for intermediate representations such as the pollutant predictions in previous time steps (which we denote by the subscript *intermediate* in our heatmaps). On the contrary, the XAI method of LRP, by design, assigns a relevance to each intermediate neuron in the network, and thus delivers also a relevance to the pollution predictions in prior time steps (which then act as an input for subsequent time steps of the forecast).

From Figure 4 we observe that the Gradient x Input statistics are the most similar to the ones from LRP (see main part of the work, Figure 10), except for the input feature station number which gets assigned an extremely high relevance. However, as we have shown in the Section "Simplifying the model with LRP", the station number could be removed from the input data without loss in prediction performance. Hence the Gradient x Input result for this feature might be misleading. When we look at the heatmap for the Saliency method, we observe that the following forecast features get assigned the most relevance: hour, wind direction and month. Additionally, for the Saliency method we plot in Figure 5 the average positive relevance of the months of the year for high O_3 (ozone) forecasts (similarly to what was previously done in Figure 1c for LRP). Here with Saliency we find that the months with the highest positive contributing relevance are January and December, which seems unplausible, since from the domain literature it is known that warm temperature and sunshine are rather linked to promote high O_3 concentration (e.g.[1–4]). So overall we find these other XAI methods to be less helpful for understanding the model´s predictions.

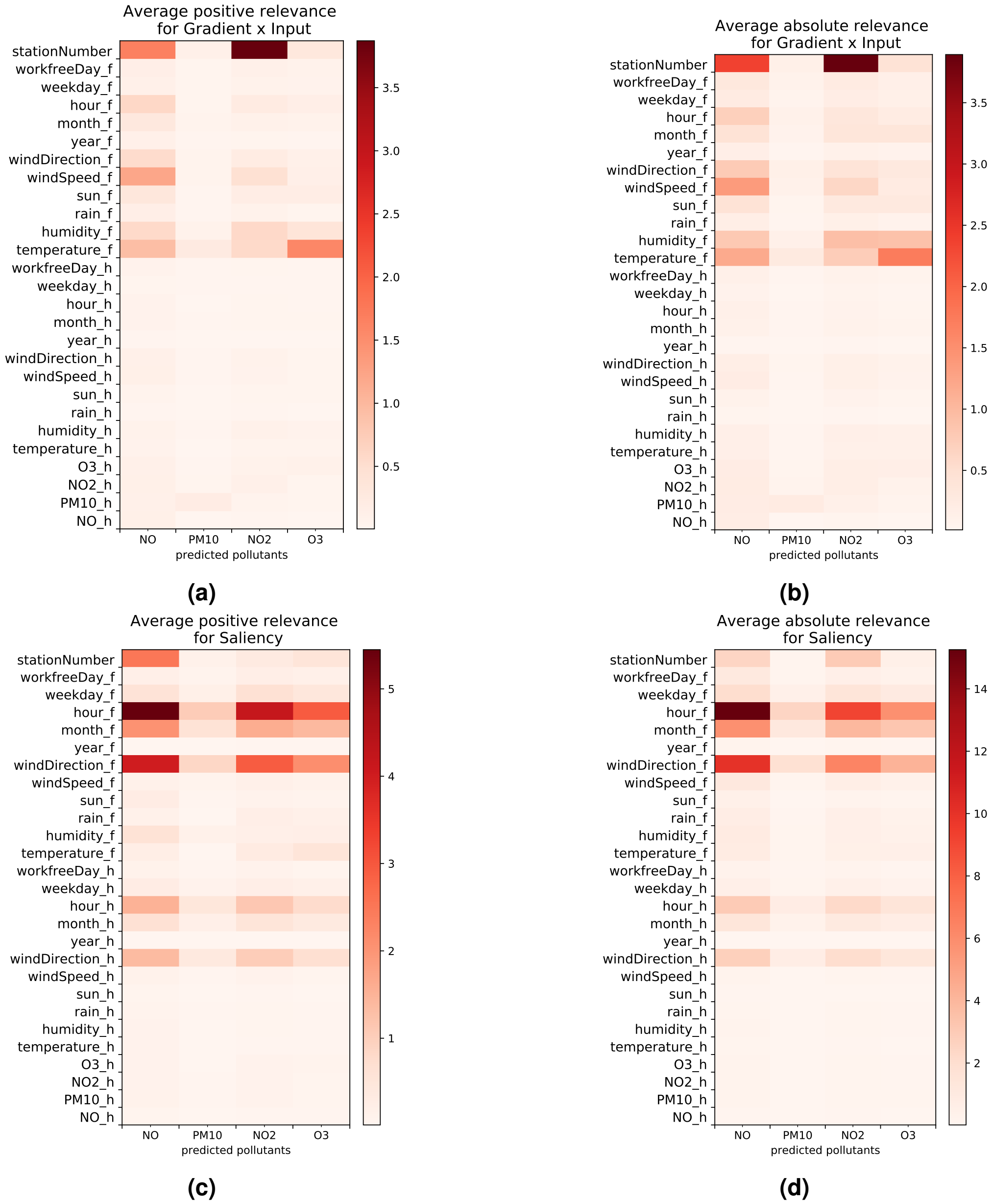

Figure 4. Average positive (left column), respectively absolute (right column), relevance for the first 24 hours of high forecasts for different pollutants and different XAI methods. Subscript *h* and *f* are used for historical and forecast input. Subscript *intermediate* is used for pollutant forecasts in previous time steps. Station number is the static input. Statistics are computed over 4,800 data points with the highest forecasts for each pollutant. [created with Matplotlib version 3.3.4 https://matplotlib.org/ and Captum version 0.4.1 https://captum.ai/]

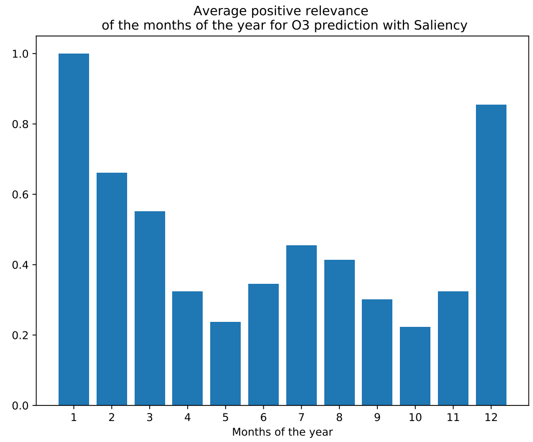

Figure 5. Average positive Saliency relevance for the input feature months of the year for high forecasts of the pollutant O_3 (quantities are rescaled such that the maximum is equal to one). Statistics are computed over 4,800 data points with the highest forecasts for this pollutant.

3 List of abbreviations and symbols

LRP .... Layer-wise Relevance Propagation

XAI .... eXplainable Artificial Intelligence

RNN .... Recurrent Neural Network

LSTM ..... Long Short-Term Memory

GRU .... Gated Recurrent Unit

PM_{10} .... Particulate Matter with diameter less than 10 μm

NO .... Nitrogen monOxide

NO_2 .... Nitrogen diOxide

NOx .... Nitrogen Oxides (sum of NO and NO_2)

O_3 .... Ozone

O_2 .... Oxygen

H_2O .... Water

HNO_3 ...... Nitric Acid

CO .... Carbon monOxide

CO_2 .... Carbon diOxide

VOCs ...... Volatil Organic Compounds

References

1. Han, S. *et al.* Analysis of the Relationship between O3, NO and NO2 in Tianjin, China. *Aerosol Air Qual. Res.* **11**, 128–139 (2011).
2. Monks, P. S. *et al.* Tropospheric ozone and its precursors from the urban to the global scale from air quality to short-lived climate forcer. *Atmospheric Chem. Phys.* **15**, 8889–8973 (2015).
3. Wang, T. *et al.* Ozone pollution in China: A review of concentrations, meteorological influences, chemical precursors, and effects. *Sci. The Total. Environ.* **575**, 1582–1596 (2017).
4. Jacob, D. J. Heterogeneous chemistry and tropospheric ozone. *Atmospheric Environ.* **34**, 2131–2159 (2000).
5. Trinh, T. T., Trinh, T. T., Le, T. T., Nguyen, T. D. H. & Tu, B. M. Temperature inversion and air pollution relationship, and its effects on human health in Hanoi City, Vietnam. *Environ. Geochem. Heal.* **41**, 929 – 937 (2019).
6. Thurston, G. D. Outdoor air pollution: Sources, atmospheric transport, and human health effects. In *International Encyclopedia of Public Health (Second Edition)*, 367–377 (2017).
7. Hörmann, S., Pfeiler, B. & Stadlober, E. Analysis and Prediction of Particulate Matter PM10 for the Winter Season in Graz. *Austrian J. Stat.* **34**, 307–326 (2005).
8. Hoyos, C. D., Herrera-Mejía, L., Roldán-Henao, N. & Isaza, A. Effects of fireworks on particulate matter concentration in a narrow valley: The case of the Medellín metropolitan area. *Environ. Monit. Assess.* **192**, 1–31 (2020).
9. Sicard, P. *et al.* Ozone weekend effect in cities: Deep insights for urban air pollution control. *Environ. Res.* **191**, 110193 (2020).
10. Arras, L., Osman, A. & Samek, W. CLEVR-XAI: A benchmark dataset for the ground truth evaluation of neural network explanations. *Inf. Fusion* **81**, 14–40 (2022).
11. Arras, L., Osman, A., Müller, K.-R. & Samek, W. Evaluating recurrent neural network explanations. In *Proceedings of the 2019 ACL Workshop BlackboxNLP: Analyzing and Interpreting Neural Networks for NLP*, 113–126 (2019).

12. Poerner, N., Roth, B. & Schütze, H. Evaluating neural network explanation methods using hybrid documents and morphosyntactic agreement. In *Proceedings of the 56th Annual Meeting of the Association for Computational Linguistics*, 340–350 (2018).

13. Mamalakis, A., Barnes, E. A. & Ebert-Uphoff, I. Investigating the Fidelity of Explainable Artificial Intelligence Methods for Applications of Convolutional Neural Networks in Geoscience. *Artif. Intell. for Earth Syst.* **1**, e220012 (2022).

14. Simonyan, K., Vedaldi, A. & Zisserman, A. Deep inside convolutional networks: Visualising image classification models and saliency maps. In *Proc of the International Conference on Learning Representations* (2014).

15. Shrikumar, A., Greenside, P., Shcherbina, A. & Kundaje, A. Not just a black box: Interpretable deep learning by propagating activation differences. *arXiv arXiv:1605.01713* (2016).

16. Kokhlikyan, N. *et al.* Captum: A unified and generic model interpretability library for PyTorch. *arXiv arXiv:2009.07896* (2020).
